# A Semantic Web Management Model for Integrative Biomedical Informatics

**DOI:** 10.1371/journal.pone.0002946

**Published:** 2008-08-13

**Authors:** Helena F. Deus, Romesh Stanislaus, Diogo F. Veiga, Carmen Behrens, Ignacio I. Wistuba, John D. Minna, Harold R. Garner, Stephen G. Swisher, Jack A. Roth, Arlene M. Correa, Bradley Broom, Kevin Coombes, Allen Chang, Lynn H. Vogel, Jonas S. Almeida

**Affiliations:** 1 Department of Bioinformatics and Computational Biology, The University of Texas M.D. Anderson Cancer Center, Houston, Texas, United States of America; 2 Instituto de Tecnologia Química e Biológica, Universidade Nova de Lisboa, Lisboa, Portugal; 3 Department of Thoracic/Head and Neck Medical Oncology, The University of Texas M.D. Anderson Cancer Center, Houston, Texas, United States of America; 4 Department of Pathology, The University of Texas M.D. Anderson Cancer Center, Houston, Texas, United States of America; 5 Hamon Center for Therapeutic Oncology Research, Simmons Cancer Center, University of Texas Southwestern Medical Center, Dallas, Texas, United States of America; 6 Department of Internal Medicine, University of Texas Southwestern Medical Center, Dallas, Texas, United States of America; 7 Eugene McDermott Center for Human Growth and Development, University of Texas Southwestern Medical Center, Dallas, Texas, United States of America; 8 Center for Biomedical Inventions, University of Texas Southwestern Medical Center, Dallas, Texas, United States of America; 9 Department of Biochemistry, University of Texas Southwestern Medical Center, Dallas, Texas, United States of America; 10 Department of Thoracic and Cardiovascular Surgery, The University of Texas M.D. Anderson Cancer Center, Houston, Texas, United States of America; 11 Department of Biomedical Informatics, Columbia University, New York, New York, United States of America; Tel Aviv University, Israel

## Abstract

**Background:**

Data, data everywhere. The diversity and magnitude of the data generated in the Life Sciences defies automated articulation among complementary efforts. The additional need in this field for managing property and access permissions compounds the difficulty very significantly. This is particularly the case when the integration involves multiple domains and disciplines, even more so when it includes clinical and high throughput molecular data.

**Methodology/Principal Findings:**

The emergence of Semantic Web technologies brings the promise of meaningful interoperation between data and analysis resources. In this report we identify a core model for biomedical Knowledge Engineering applications and demonstrate how this new technology can be used to weave a management model where multiple intertwined data structures can be hosted and managed by multiple authorities in a distributed management infrastructure. Specifically, the demonstration is performed by linking data sources associated with the Lung Cancer SPORE awarded to The University of Texas MDAnderson Cancer Center at Houston and the Southwestern Medical Center at Dallas. A software prototype, available with open source at www.s3db.org, was developed and its proposed design has been made publicly available as an open source instrument for shared, distributed data management.

**Conclusions/Significance:**

The Semantic Web technologies have the potential to addresses the need for distributed and evolvable representations that are critical for systems Biology and translational biomedical research. As this technology is incorporated into application development we can expect that both general purpose productivity software and domain specific software installed on our personal computers will become increasingly integrated with the relevant remote resources. In this scenario, the acquisition of a new dataset should automatically trigger the delegation of its analysis.

## Introduction

### Data management and analysis for the life sciences

**Figure 1:**
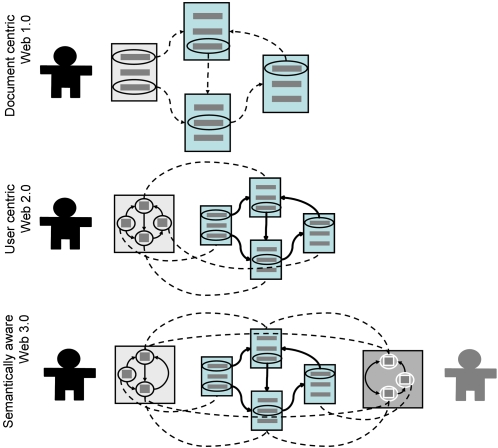
Three generations of design patterns for web-based applications. The original design (“1.0”) consists of collections of hypertext documents that are syntactically (dashed lines) interoperable (traversing between them by clicking on the links), regardless of the domain content. The user centric web 2.0 applications use internal representations of the external data structures. This representation is asynchronously updated from the reference resources which are now free to have a specialized interoperation between domain contents. An example of this approach is that followed by AJAX-based interfaces. Finally, the ongoing emergence of the semantic web promises to produce service oriented systems that are semantically interoperable such that the interface application reacts to domains of knowledge specifically. At this level all applications tend to be web-interoperable with peer-to-peer architectures complementing the client-server design of w1.0 and w2.0.

“The laws of Nature are written in the language of mathematics” famously said Galileo. However, in recent years efforts to analyze the increasing amount and diversity of data in the Life Sciences has been correspondingly constrained not so much by our ability to read it as by the challenge of organizing it. The urgency of this task and the reward of even partial success in its accomplishment have caused the interoperability between diverse digital representations to take center stage [1]–[5]. Presently, for those in the Life Sciences enticed by Galileo's pronouncement, the effort of collecting data is no longer focused solely on field/bench work. Instead, it often consists of painfully squeezing the pieces of the systemic puzzle from the digital media where the raw data is held hostage[6]. It is only then that a comprehensive representation amenable to mathematical modeling really becomes available[7]. This is not a preoccupation exclusive to the Life Sciences. Integration of software applications is also the driving force behind new information management systems architectures that seek to eliminate the boundaries to interoperability between data and services. This preoccupation indeed underlies the emergence of service oriented architectures [8]–[11], even more so in its event driven dynamic generalization [12]. It also underlies the development of novel approaches to software deployment (Figure 1) that juggle data structures between server and client applications. Presently, a particularly popular design pattern is the usage-centric Web 2.0 [13], [14] which seeks a delicate balance in the distribution of tasks between client and server in order to diminish the perception of a distinction between local and remote computation.

Semantic web technologies [3], [15]–[21] represent the latest installment of web technology development. In what is being unimaginatively designated as Web 3.0[22], [23], a software development design pattern is proposed where the interoperability boundaries between data structures, not just between the systems that produce them, is set to disappear. The defining characteristic of this environment is that one can retrieve data and information by specifying their desired properties instead of explicitly (syntactically) specifying their physical location. The desirability of this design can clearly be seen in systems in which clinical records are matched with high throughput molecular profiles, each of which stem from very distinct environments and are often the object of very different access management regulations.

### Inadequacy of conventional systems for Translational Research

On the one hand, high throughput molecular Biology core facilities and improved medical record systems are able to document individual data elements with increasing detail. On the other hand, researchers producing the data and models that critically advance the understanding of biological phenomena are increasingly separated from their use by the specialization inherent in each of these activities. Consequently, bridging between the information systems of basic research and their clinical application becomes a necessary foundation for any translational exploits of new biomedical knowledge[3], [24]. The alternative, using conventional data representations where the data models cannot evolve, typically requires the biomedical community to complement the data representation with a clandestine and inefficient flurry of datasets exchanged as spreadsheets through email.

### Foundations for a novel solution

As others before us[5], we have argued previously for the use of semantic web formats as the foundation for developing more flexible and articulated data management and analytical bioinformatics infrastructures[20]. A software prototype was then produced following those technical specifications to provide a flexible web-based data sharing environment within which a management model can be identified[24]. In this third report we describe the resulting core model supporting distributed and portable data representation and management. In practice this translates into a small application deployed in multiple locations rather than a large infrastructure at a single central location. The open source prototype application described here has been made public[25]. All deployments support a common data management and analysis infrastructure with no constraints on the actual data structures described.

### A very brief history of data

**Figure 2:**
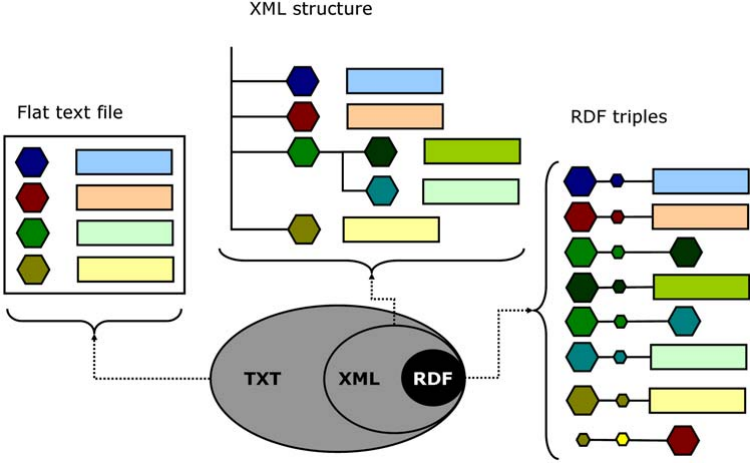
Evolution of formats for individual datasets. Hexagons, rectangles and small circles indicate data elements, respectively, attributes, their values, and relations. First, flat file formats such as fasta or the GeneBank data model were proposed to collect attribute-value pairs about an individual data entry. The use of tagging by extended markup languages (XML) allowed for the embedding of additional detail and further definition of the nature of the hierarchical structure between data elements. More recently, the resource description framework (RDF) further generalized the XML tree structure into that of a network where the relationship between resources (nodes) is a resource itself. Furthermore, the referencing of each resource by a unique identifier (URI) implies that the data elements can be distributed between distinct documents or even locations.

The formatting of data sets as portable text mirrors the same three stages described for web-based applications in Figure 1. As described in Figure 2, data representation has been evolving from tabular text formats (“flat files”), to self described hierarchical trees of tags (extended markup languages, XML), and finally to the subject-predicate-object triples of Resource Description Framework (RDF)[26]. We have been active participants in these transformations [24], [27], [28], and like many others concluded that in order to bridge the fragmentation between distinct data structures, we needed to break down the data structures themselves[20], that is, to reduce the interoperable elements to RDF triples[29]. In addition to its directed labeled graph nature, RDF formats[29] have a second defining characteristic: each of the three elements has a Uniform Resource Identifier (URI), which, for the purposes of this very brief introduction, can be thought as a unique locator capable of directing an application to the desired content or service. It is also interesting to note that at each level of this three-stage progression (Figure 2) we find data elements that have “matured”, that is, that present a stable representation which remains useful to specialized tools. When this happens we find that those elements remain convenient representations preserved whole within more fragmented formats. For example, we find no advantages in breaking down mzXML[30] representations of mass spectrometry based proteomics data. Instead, these data structures are used as objects of regular RDF triples. The mzXML proteomics data structure offers an paradigmatic illustration of the evolution of ontologies as efforts to standardize data formats[31]. It would be interesting to understand if the lengthy effort headed by the Human Proteomics Organization, HUPO, to integrate it reflects the difficulty to justify reforming[32] a representation that remains useful[33].

The advancement towards a more abstract, more global and more flexible representation of data is by no means unique to the Life Sciences. However, because of the exceptional diversity of that domain's fluidity, the Life Sciences are where the Semantic Web may find its most interesting challenge and as well, hopefully, where it will find its most compelling validation[15].

### Mathematics for data models

It has not been lost to the swelling ranks of Systems Biologists that the reduction of data interoperability to the ternary representation of *relations* [34] brings the topic solidly back to the Galilean fold of Mathematics as a language. The reduction of data structures to globally referenced dyadic relations (functions of two variables), such as those of the Entity-Relationship (ER) model, brings in rich feeds from the vein of Logic. In the process, and beyond Galileo's horizon, assigning a description logic value[35]–[37] to some RDF predicates (for example, specifying that something is part of or, on the contrary, is distinct from something else) allows the definition of procedures. This further elaboration of RDF has the potential to transform data management into an application of knowledge engineering, and more specifically of artificial intelligence (AI). This reclassification reflects the dilution of the distinction between data management and data analysis that is apparent even in an introduction as brief as this one. Another clear indication of this transformation is that it re-ignites the opposition between data-driven and rule-driven designs for semantic web representation[38]–[42], a recurring topic in AI. It is important to note that the management model proposed here is orthogonal to that discussion. Its purpose is solely to enable the distribution[43] of a semantic data management system that can withstand changes in the domain of discourse, independently of the rationale for the changes themselves.

### Software engineering for Bioinformatics

This overview of modern trends in integrative data management is as significant for what is covered as for what is missed – what management models should be used to control the generation and transformation of the data model? It is interesting to note that the management models that associate access permissions with the population of a data model have traditionally been the province of software engineering. This may at first appear to be a reasonable solution. Since instances of a data structure in conventional databases are contained in a defined digital media, permission management is an issue of access to the system itself. However, this ceases to be the case with the semantic web RDF triples because they weave data structures that can expand indefinitely between multiple machines. Presently, the formalisms to manage data in the semantic web realm are still in the early stages of development, notably by the World Wide Web consortium (W3C) SKOS initiative (Simple Knowledge Organization Systems). This initiative recently issued a call[44] for user cases where good design criteria can be abstracted and recommendations be issued on standard formats. As expected[15], the Life Sciences present some of the most convoluted user cases in which a multitude of naïve domain experts effectively need to maintain data structures that are as diverse and fluid as the experimental evidence they describe[24].

## Materials and Methods

The most extreme combination of heterogeneous data structures and the need for very tight control of access is arguably found in applications to Personalized Medicine, such as those emerging for cancer treatment and prevention. At the Univ. Texas MDAnderson Cancer Center at Houston and the Southwestern Medical Center at Dallas we have deployed the S3DB semantic web prototype to engage the community of translational researchers of the University of Texas Lung Cancer SPORE [45] in identifying a suitable management model. This exercise involved over one hundred researchers and close to half a million data entries, of clinical and molecular nature. Right at its onset integrating access permissions in the definition of the data models was identified as an absolute necessity by the participants, as anticipated by the SKOS group. As a consequence, a data driven “core model”, S3DBcore, that accommodates management specifications as part of data representation, was developed and is described here. The software used is provided with open source at www.s3db.org. Only open source tools were used in development of this web-based web-service: PHP 5 was used for server side programming and both MySQL and PostgreSQL were tested as the relational backbone for PHP's database abstraction class. At the same location detailed documentation about S3DB's Application Programming Interface (API) is also provided.

## Results

### Units of representation

**Figure 3:**
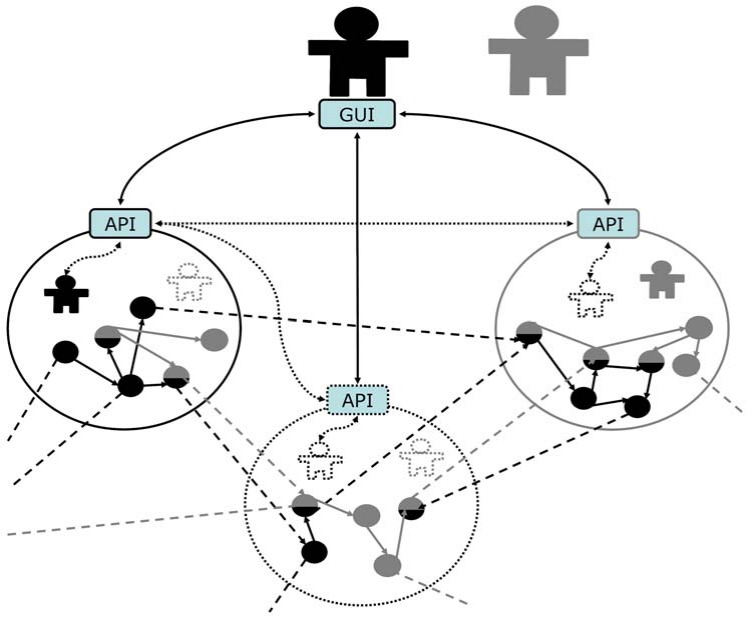
Illustration of the desirable functionality: distinct users, with identities (solid icon) managed in distinct S3DB deployments (circular compartments), which they control separately, share a distributed and overlapping data structure (arrows between symbols) that they also manage independently: some data elements are shared (mixed color symbols) others are not. This will require the identity verification to propagate between deployments peer-to-peer (P2P, dotted lines), including to deployments where neither user maintains an identity (dotted circular compartment). This is in contrast with the conventional approach of having distinct users manage insular deployments with permissions managed at the access point level.

The most fundamental representation of data is that of attribute-value (AV) pairs, for example, <color,”blue”>. The generic data management infrastructure proposed here can be described as that of encapsulating AV pairs through the use of another fundamental unit of representation, the Entity-Relation-Entity model (ER), such as <sky, has, color>. Each entity can then be associated with one or more AV pairs using the entity-attribute-value EAV model, for example, <sky, color, ”blue”>. Fast forwarding three decades of computer science and knowledge engineering and we reach the present day development of a representation framework where each element of the triple is a resource with a unique identifier, with the third element of the triple having the option of being a literal, that is, of having an actual value rather than a placeholder. This single sentence very broadly describes the Resource Description Framework (RDF) which is at the foundation of the ongoing development of the Semantic Web[29], just like hypertext (HTML) was the enabling format for the original Web. It is important to note that the evolution of representation formats typically takes place through generalization of the existing ones. For example, extended markup language-based files (XML) are still text files, and RDF documents are still XML structures (Figure 2). As noted earlier, this succession is closely paralleled by refinements of software design patterns (Figure 1). This reification process is often driven by the necessity to maintain increasingly complex data at a simpler level of representation where they remain intelligible for those who generate and use the data. Accordingly, in the next section triple relations will be weaved around the AV pair with that exact purpose: to produce a core model that is simple enough to be usable by naïve users that need to interact with heterogeneous data hosted in a variety of machines (Figure 3), yet sophisticated enough to support automated implementation.

### Weaving a distributed information management system

**Figure 4:**
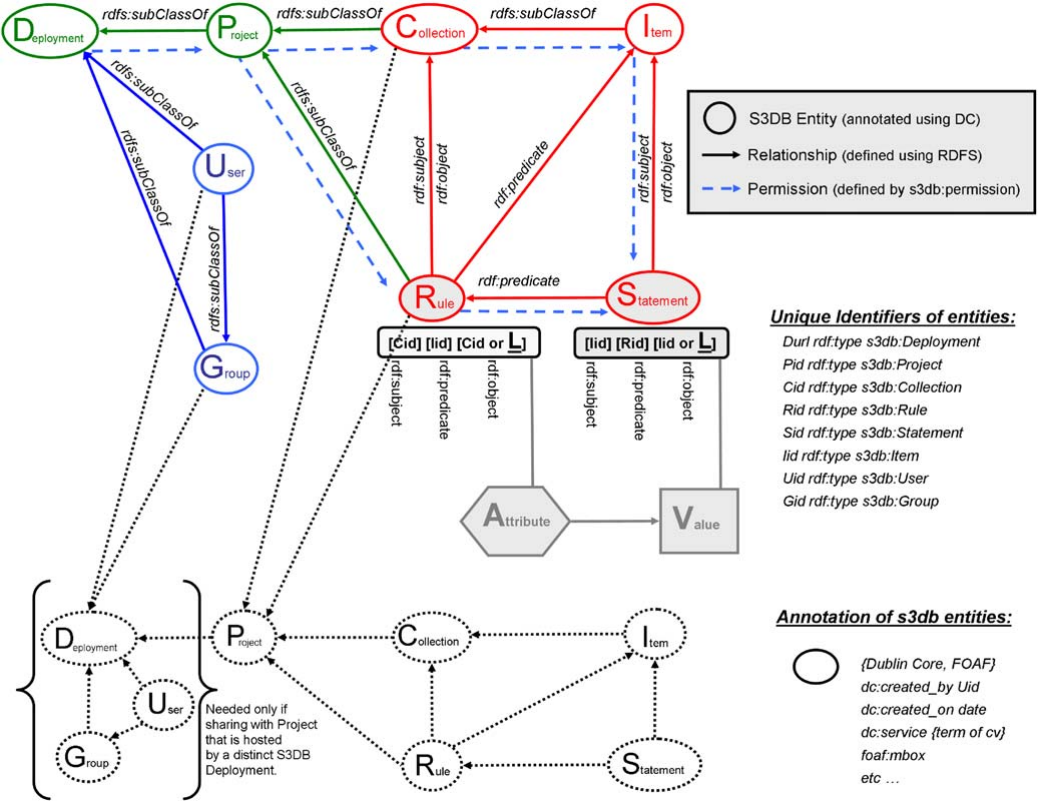
Core model developed for S3DB (supported by version 3.0 onwards). This diagram can be read starting from the most fundamental data unit, the Attribute-Value pair (filled hexagonal and square symbols). Each element of the pair is object of two distinct triples, one describing the domain of discourse, the *Rules*, and the other made of *Statements* where that domain is populated to instantiate relationships between entities. The latter includes the actual Values. Surrounding these two nuclear collection of triples, is the resolution of *Collection* and its instantiation as *Item* that define the relationship between the individual elements of *Rules* and *Statements*. The resulting structure is then organized in *Projects* in such a way that the domain of discourse can nevertheless be shared with other *Projects*, in the same or in a distinct deployment of S3DB. Finally, a propagation of user permissions (dashed line) is defined such that the distribution of the data structures can be traced. See text for a more detailed description.

The objective of this exercise is to produce a data management model that can be distributed through multiple deployments of the Database Management Systems (DBMS) which implies a mechanism for migration access permissions. Simultaneously, this model should allow different domain experts to evolve their own data models without compromising pre-existing data. Achieving these two goals simultaneously can only be realized if the proposed distributed system is composed of node applications that are not only syntactically interoperable, but also semantically transparent. For a discussion of the absolute need for evolvable data models in the Life Sciences see [24]. That report is also where the DBMS prototype, S3DB, was first introduced (version 1.0). Finally, the Application Programming Interface (API) needs to support the semantic interoperability in a way that spans multiple deployments (Figure 3). The data model developed to achieve these goals is described in Figure 4.

### A Core data management model that is universal and distributed

The directed labeled graph nature of RDF triples, coupled with their reliance on unique identifiers (as URIs), enables data structures to be scattered between multiple machines while permitting different domains of discourse to use the same data elements differently. However, those two characteristics alone do not address the management issue: how to decide when, where and what can be viewed, inserted, deleted and by whom. It is clear that the conventional approach of dealing with permissions at the level of access to the data store is not appropriate to the Life Sciences[5] where multiple disciplines and facilities are contributing to a partially overlapping representation of the system. It cannot be overstated that this is particularly the case when the system is designed to host clinical data. To solve this problem we have developed a core data model where membership and permission can migrate with the data. We have also developed a prototype application to support such a distributed data management system (Figure 3), which we make freely available with open source[25].

## Discussion

The proposed core model is detailed in Figure 4 and will be now discussed in more detail. This diagram is best understood chronologically, starting with the very basic and nuclear collection of attribute-value pairs and then proceeding to their encapsulation by three consecutive layers – the semantic schema, assignment of membership and, finally the permission propagation.

### Schema

The first layer of encapsulation is the definition and use of a domain of discourse (elements in red in Figure 4). This was achieved in typical RDF fashion by defining two sets of triples, one defining a set of rules and the second, the statements, using them. As discussed elsewhere [24], there are good reasons to equip those who generate the data with the tools to define and manage their own domains of knowledge. The ensuing incubation of experimental ontologies was facilitated by an indexing scheme that mimics the use of subject, verb, object in natural languages. This indexing is achieved by recognizing *Collections* and the *Items* they contain as elements of the two sets of nuclear triples (*Rules* and *Statements*).

### Organization

The second layer of formal encapsulation corresponds to the assignment of membership. This process extends the designation of *Items* in the previous level, by assigning the *Collections* that contain them and *Rules* that relate them to *Projects* that are hosted by individual *Deployments* of the prototype S3DB application. In the diagram, the membership dependencies are accordingly labeled as *rdfs*∶*subClassOf* [29]. Note that memberships can also be established with remote resources (dotted lines in Figure 4), that is, between resources of distinct deployments. Defining remote memberships presents little dificulty in the RDF format because each element of the triple is refered to by a universal identifier (a URI), unique accross deployments. On the other hand, managing permission to access the remote content is a much harder problem, which we will address by supporting migration of identity. The alternative solution to migration of identities is migrating the contents along membership lines. However, that was, unsurprisingly, found to be objectionable by users with a special attention to privacy and confidentiality issues. It would also present some logistic challenges for larger datasets. In contrast, the definition of a temporary, portable, identity key or token needed for migration of identity is typically incommensurably smaller than the content it permits access.

### Permissions

The final layer of encapsulation defines *Users* and *Groups* within *Deployments* and controls their permissions to the data (blue in Figure 4). As with rest of the core model, the identification of proposed management of permissions was directed by user cases. That exercise determined that user identities should be maintained by specific Deployments of S3DB but also that they may be temporarily propagated to other deployments. That solution, illustrated in Figure 3, allows one application to request the verification of an identity in a remote deployment, which then verifies it in the identity's source deployment and assigns it a temporary key or token, say, for one hour. All that is propagated is a unique alphanumeric string, the temporary token, paired with the user's URI. No other user information is exchanged. As a consequence, for the remainder of the hour, the identification will be asynchronously available in both deployments, which enables the solution described in Figure 3, where a single interface can manipulate multiple components of a large, distributed systems level representation of the target data. Interestingly, because the multiple deployments of S3DB are accessed independently by multiple deployments of various applications, the mode of syntactic interoperation is *de facto* peer-to-peer. The propagation of permissions flows in the sequence indicated by the dashed blue lines in Figure 4. When a permission level is not defined for a resource, say for a *Item*, then it is borrowed from the parent entity, in this example, from the corresponding *Collection*. When there is a conflict then the most restrictive option is selected. For example a conflict can arise for a *Statemen*t which inherits permissions from both *Rules* and *Collections*. Another frequent example happens when a user belongs to multiple groups with distinct permissions to a common target resource.

Permission management is a particularly thorny issue in life sciences applications because of the management of multiple data provenances. Relying on distributed hosting of the complementary data sources compounds the management of multiple permissions even further because it also involves multiple permission management systems. Finally, permission management is often treated *ad hoc* by the management systems themselves where it is resolved as access permission to the system as a whole rather than being specified in the data representation. Because each source often describes a specialized domain, it is guarded with understandable zeal. We argue here that propagation of permissions is the only practical solution to determine how much information is to be revealed in different contexts. Consequently, whereas the relationships between the 8 S3DB entities (oval symbols in Figure 4) are defined using RDF schema[26] (RDFS), and their tagging uses the well established Dublin Core[46], the permission propagation layer is a novel component of the proposed management model. In order to respond to widest range of the user cases driving model identification, the propagation was defined by three parameters, view, edit, and use. Each of these parameters can have three values, 0, 1 or 2, corresponding to, respectively, no permission, permission only on entries submitted by the user, and permission on all entries of that resource. *Users* and *Groups* (blue entities in Figure 4) can have these three types of permissions on *Projects*, *Collections*, *Rules*, *Items* and *Statements*. Among those five entities, additional permissions can be issued, for example, a *Project* may have specific permissions on *Collections* and *Rules*. *Collections* may have further permissions on their *Items*. The same reasoning, in reverse, establishes what should happen when permission is not specifically defined for a given entity. For example, for a *Statement* the permission would be inherited from the parent entities, *Item* and *Rule*. If those two entities did not specify specific permissions for the target statement, then those are searched upstream (Figure 4) until reaching the *Project* or even *Deployment* level. According to this mechanism, the conventional role of a system administrator corresponds to a user with permissions 222 at Deployment level. It is worth recalling that propagation of permissions between data elements in distinct S3DB deployments happens through the sharing the membership in external *Collections* and *Rules* (dotted lines), not through extending the permission inheritance beyond the local deployment. This is not a behavior explicitly imposed on the distributed deployment; it emerges naturally from the fact that *Rule* sharing specifies a permission which, remote or local, interrupts the permission inheritance. In practice both the user of the interface and the programmer using the API can ignore the intricacies of this process, which was identified to be the intuitive, sensible, propagation of permissions that we found naïve users to expect in user-case exercises.

### Portability

This discussion would not be complete without unveiling some defining technical details about how portability is addressed by this design. So far we have been loosely equating “unique identifiers” with the use of Uniform Resource Identifiers (URI). More specifically, the right hand side of Figure 4 includes a list of eight types of locally unique identifiers that can be assigned to the same number of entities that define the core model. It is easy to see how this indexing can be made globally unique by concatenating them with the *Deployment*'s ID, itself unique, for example using its URL. Indeed this is what is supported by the accompanying prototype software, with a generalizing twist with very significant consequences: *Did* can either be the deployment address or anything that indicates what that address is. For example, it can indicate an HTML document or even an entry in a database where this address is specified. More interestingly, it can also be a simple alphanumeric code that is maintained at www.s3db.org in association with the actual URL of the target deployment. The flexible global indexing achieved by either scenario allows the manipulation of entire databases management systems as portable data structures. It also allows for novel management solutions through manipulation of the DBMS logical structure. For example, defining a *Did* as ‘localhost’ would have the effect of severing all logical connections to any usage outside that of the server machine. None of these more fanciful configurations were validated with the Lung Cancer SPORE user community even if they are fully supported by the accompanying prototype. Nevertheless, its possibility enables some interesting scenarios for data management and indeed for Knowledge Engineering.

### User Interfaces

**Figure 5:**
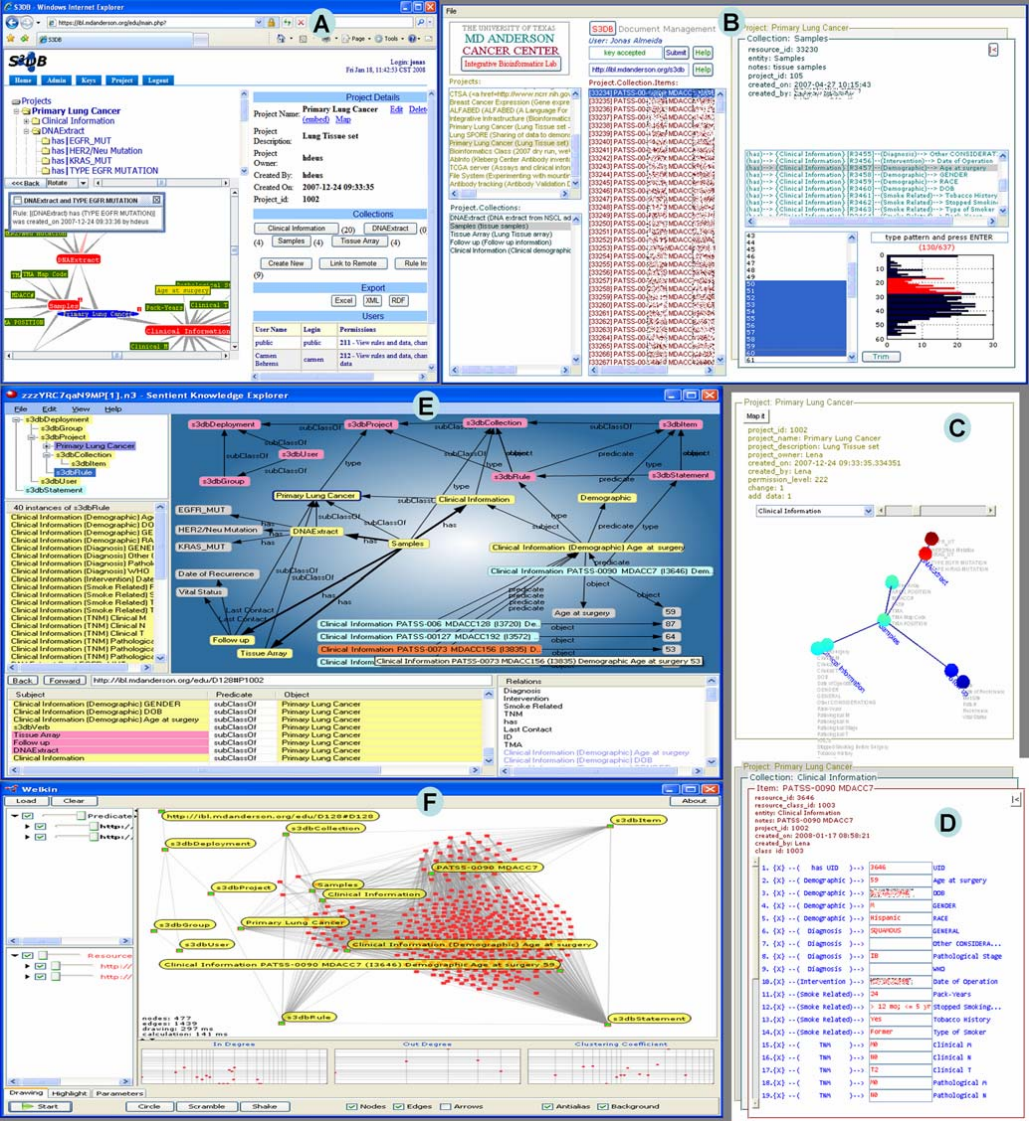
Snapshots of interfaces using S3DB's API (Application Programming Interface). These applications exemplify why the semantic web designs can be particularly effective at enabling generic tools to assist users in exploring data documenting very specific and very complex relationships. Snapshot A was taken from S3DB's web interface, which is included in the downloadable package[25]. This interface was developed to assist in managing the database model and, therefore, is centered on the visualization and manipulation of the domain of discourse, its *Collections of Items* and *Rules* defining the documentation of their relations. The application depicted on snapshots B–D describe a document management tool S3DBdoc, freely available as a Bioinformatics Station module (see Figure 6). The navigation is performed starting from the Project (C), then to the *Collection* (B) and finally to the editing of the *Statements* about an *Item* (D). The snapshot B illustrates an intermediate step in the navigation where the list of *Items* (in this case samples assayed by tissue arrays, for which there is clinical information about the donor) is being trimmed according to the properties of a distant entity, Age at Diagnosis, which is a property of the Clinical Information *Collection* associated with the sample that originated the array results. This interaction would have been difficult and computationally intensive to manage using a relational architecture. The RDF formatted query result produced by the API was also visualized using a commercial tool, Sentient Knowledge Explorer (IO-Informatics Inc), shown in snapshot E, and by Welkin, developed by the digital inter-operability SIMILE project at the Massachusetts Institute of Technology. See text for discussion of graphic representations by these tools. To protect patient confidentiality some values in snapshots B and D are scrambled and numeric sample and patient identifiers elsewhere are altered.

**Figure 6:**
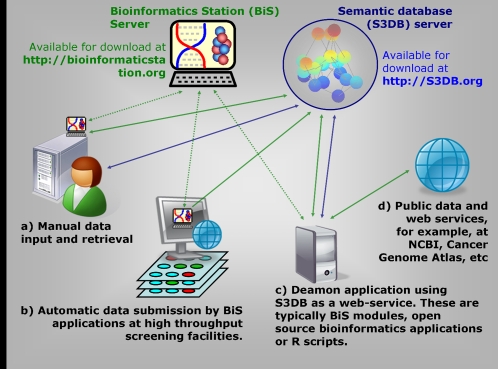
Prototype infrastructure for integrated data management and analysis being tested by the Univ. Texas Lung cancer SPORE. The system is based on two components, a network of universal semantic database servers and a code distribution server that delivers applications in response to the use of ontology. Four distinct user cases are represented, a–d, which rely on a combination of download of interpreted code (green arrows) or direct access to web-based graphic user interfaces or web-based API (blue arrows, in the latter case using Representational State Transfer, REST). The dotted lines represent regular updating of the application, propagating improvements in the application code.

The ultimate test for a data management model is the intuitiveness of what it communicates through the user interface[47], [48]. The structure of S3DBcore offers some useful guidelines in this regard. The experimental values are represented in a combination of *Items* and *Statements* (Figure 4). There are two routes to that endpoint. One possibility is to take the document management approach of navigating from *Projects* to *Collections*, then to their *Items* and finally to the *Statements*. This is the scenario that will suit data centric activities such as querying and updating existing data or inserting new data. A real, working example of how that interface may look is depicted in Figure 5-B, which details an intermediate step between selecting a *Project* (Figure 5-B), and identifying and manipulating an individual entry made of multiple statements about an *Item* (Fig. 5-D). The mechanism used to distribute rich graphics applications and their interoperation with S3DB is detailed in Figure 6. Another possibility is to navigate from the *Project* to the collection of *Rules*, most likely represented as a directed labeled graph network, and then browse the *Statements* as an instantiation of the *Rules*, exemplified by another snapshot of a working application, Figure 5-A. This application is the standard web-based user interface distributed with S3DB package[25]. Unlike the bookkeeping approach of the document centric model (Figure 5-B), the rule centric view (Figure 5-A) is most suitable to investigate the relationship between different parts of the domain of knowledge and to incubate[24] a more comprehensive and exact version of the ontology. However, and this may be the most relevant point, since S3DB's API returns query results as RDF, any RDF browser can be used to explore it. This point is illustrated in figures 5E and F where, respectively, a commercial semantic web knowledge explorer (Sentient, IO-Informatics Inc) and Welkin, a popular RDF browser developed at the Massachusetts Institute of Technology, are use to visualize the same S3DB Lung Cancer project depicted in Figs. 5A and B. Whereas the former is designed as a tool for knowledge discovery, the latter offers a global view of distributed data structures. The value of the core model described in Figure 4 as a management template for individual data elements will be apparent upon close inspection of Fig. 5E. The different colors, automatically set by Sentient KE, distinguish the core model (pink), where permission management takes place, from the instantiation of their entities, in yellow. These two layers describe the context for individual entries specifying the age at surgery of 5 patients. The same display includes access to molecular work on tumor samples, in this case using tissue arrays and DNA extracts. The distinct domains are therefore integrated in an interoperable framework in spite of the fact that they are maintained, and regularly edited, by different communities of researchers. As a consequence, the database can evolve with the diversification of data gathering methodologies and with the advancement in understanding the underlying processes. In figure 5F it can be seen that MIT's Welkin RDF visualizer easily distinguished the query results as the interplay of 4 collections of 380 *Statements* about 41 *Items* from 5 *Collections* related by 40 *Rules*. For comparison, see Figure 5E where one of its *Statements* is labeled (describing that Age of patient providing pathology sample #90 with Clinical Information #I3646 is 90 years old), along with the parent entities. For examples of other *Statements* about the same *Item* see Fig. 5D. For examples of other statements of the same nature (about the same domain), see 4 statements listed at the bottom-right of Figure 5E.

### Conclusion

The Semantic Web[15] technologies have the potential to addresses the need for distributed and evolvable representations that are critical for systems Biology and translational biomedical research. As this technology is incorporated into application development we can expect that both general purpose productivity software and domain specific software installed on our personal computers will become increasingly integrated with the relevant remote resources. In this scenario, the acquisition of a new dataset should automatically trigger the delegation of its analysis. The relevance of this achievement becomes very clear when we note that what prevents a new microarray result from being of immediate use to the experimental Biologist acquiring it is not the computational capability of the experimentalist's machine. Biostatisticians do not necessarily have more powerful machines than molecular Biologists. Moreover, in neither case is high end computation expected to be performed in the client machine[8]. Rather, once data gathering and data analysis applications become semantically interoperable, at the very least, those who acquire the illustrative microarray data should expect their own machines to automatically trigger its sensible analysis by background subtraction, normalization and basic multivariate exploratory analysis such as dimensionality reduction and clustering. As a consequence, the quantitative scientist's role can be focused on defining the sensibility of alternative contexts of data generation.

The consequences of semantic integration are just as advantageous for those dedicated to data analysis. Statistical analysts typically spend the majority of their time parsing raw datasets rather than assessing the reasonableness of alternative analytical routes. This contrasts with the critical need to validate any given analysis by comparing results produced by alternative configurations applied to independent experimental evidence. It is this final step that ultimately determines the sensibility of the data analysis procedures triggered by the acquisition of data. In summary, any data management and analysis system that will scale for systems level analysis in the Life Sciences has to be semantically interoperable if automated validation is to be attainable.

In this report, we have demonstrated the design of a semantic web data model, S3DBcore, capable of delivering the desired features of distribution and evolvability. This solution relies on RDF triples, the language developed to enable the semantic web in the same fashion that HTML was developed to enable the original web. However, collections of *subject-predicte-object* triples do not establish a management model by themselves. That exercise requires the encapsulation of the data within two additional layers, one confining membership and another permitting access. The effort of identifying management models for information systems has conventionally been the property of technology deployment. This is not feasible when the challenge is scaled to the level of complexity and distribution of Systems Biology. This report describes such a working management model and the authors also make its prototype deployment freely available with open source. In conclusion, a distributed integrated data management and analysis system might look like the prototype infrastructure described in Figure 6 which is based on a semantic database backbone coupled to a code distribution server reacting to the domain of discourse being used.

## References

[1] Blake JA, Bult CJ (2006). Beyond the data deluge: data integration and bio-ontologies.. J Biomed Inform.

[2] Komatsoulis GA, Warzel DB, Hartel FW, Shanbhag K, Chilukuri R (2007). caCORE version 3: Implementation of a model driven, service-oriented architecture for semantic interoperability.. J Biomed Inform.

[3] Ruttenberg A, Clark T, Bug W, Samwald M, Bodenreider O (2007). Advancing translational research with the Semantic Web.. BMC Bioinformatics.

[4] Brazhnik O, Jones JF (2007). Anatomy of data integration.. J Biomed Inform.

[5] Hendler J (2003). Communication. Science and the semantic web.. Science.

[6] Wiley HS, Michaels GS (2004). Should software hold data hostage?. Nat Biotechnol.

[7] Wass J (2006). Integrating Knowledge.. Bio-IT World.

[8] Foster I (2005). Service-oriented science.. Science.

[9] Hey T, Trefethen AE (2005). Cyberinfrastructure for e-Science.. Science.

[10] Nadkarni PM, Miller RA (2007). Service-oriented architecture in medical software: promises and perils.. J Am Med Inform Assoc.

[11] Bridges MW (2007). SOA in healthcare, Sharing system resources while enhancing interoperability within and between healthcare organizations with service-oriented architecture.. Health Manag Technol.

[12] Gomadam R, Ramaswamy, Sheth, Verma (2007). A Semantic Framework for Identifying Events in a Service Oriented Architecture.. IEEE International Conference on Web Services ICWS.

[13] Musser J, O'Reilly T (2006). Web 2.0 Principles and Best Practices;.

[14] Kamel Boulos MN, Wheeler S (2007). The emerging Web 2.0 social software: an enabling suite of sociable technologies in health and health care education.. Health Info Libr J.

[15] Berners-Lee T, Hall W, Hendler J, Shadbolt N, Weitzner DJ (2006). Computer science. Creating a science of the Web.. Science.

[16] Berners-Lee T, Hendler J (2001). Publishing on the semantic web.. Nature.

[17] Gordon PM, Trinh Q, Sensen CW (2007). Semantic Web Service provision: a realistic framework for Bioinformatics programmers.. Bioinformatics.

[18] Neumann E, Prusak L (2007). Knowledge networks in the age of the Semantic Web.. Brief Bioinform.

[19] Post LJ, Roos M, Marshall MS, Driel RV, Breit TM (2007). A semantic web approach applied to integrative bioinformatics experimentation: a biological use case with genomics data.. Bioinformatics.

[20] Wang X, Gorlitsky R, Almeida JS (2005). From XML to RDF: how semantic web technologies will change the design of ‘omic’ standards.. Nat Biotechnol.

[21] Feigenbaum L, Martin S, Roy MN, Szekely B, Yung WC (2007). Boca: an open-source RDF store for building Semantic Web applications.. Brief Bioinform.

[22] Borland J (2007). A Smarter Web.. Technology Review March/April.

[23] Green H (2007). A Web That Thinks Like You.. Businessweek.

[24] Almeida JS, Chen C, Gorlitsky R, Stanislaus R, Aires-de-Sousa M (2006). Data integration gets ‘Sloppy’.. Nat Biotechnol.

[25] s3db 2.0

[26] Robu I, Robu V, Thirion B (2006). An introduction to the Semantic Web for health sciences librarians.. J Med Libr Assoc.

[27] Silva S, Gouveia-Oliveira R, Maretzek A, Carrico J, Gudnason T (2003). EURISWEB–Web-based epidemiological surveillance of antibiotic-resistant pneumococci in day care centers.. BMC Med Inform Decis Mak.

[28] Stanislaus R, Chen C, Franklin J, Arthur J, Almeida JS (2005). AGML Central: web based gel proteomic infrastructure.. Bioinformatics.

[29] Ivan Herman RS, Dan Brickley (2007). Resource Description Framework (RDF).. The World Wide Web Consortium.

[30] Pedrioli PG, Eng JK, Hubley R, Vogelzang M, Deutsch EW (2004). A common open representation of mass spectrometry data and its application to proteomics research.. Nat Biotechnol.

[31] Orchard S, Jones AR, Stephan C, Binz PA (2007). The HUPO pre-congress Proteomics Standards Initiative workshop. HUPO 5th annual World Congress. Long Beach, CA, USA 28 October-1 November 2006.. Proteomics.

[32] Orchard S, Montechi-Palazzi L, Deutsch EW, Binz PA, Jones AR (2007). Five years of progress in the Standardization of Proteomics Data 4(th) Annual Spring Workshop of the HUPO-Proteomics Standards Initiative April 23–25, 2007 Ecole Nationale Superieure (ENS), Lyon, France.. Proteomics.

[33] Klimek J, Eddes JS, Hohmann L, Jackson J, Peterson A (2007). The Standard Protein Mix Database: A Diverse Data Set To Assist in the Production of Improved Peptide and Protein Identification Software Tools.. J Proteome Res.

[34] Aho JDU AV (1979). Universality of data retrieval languages.. Proceedings of the 6th ACM SIGACT-SIGPLAN symposium on Principles of programming languages.

[35] Aranguren ME, Bechhofer S, Lord P, Sattler U, Stevens R (2007). Understanding and using the meaning of statements in a bio-ontology: recasting the Gene Ontology in OWL.. BMC Bioinformatics.

[36] Lam HY, Marenco L, Shepherd GM, Miller PL, Cheung KH (2006). Using web ontology language to integrate heterogeneous databases in the neurosciences.. AMIA Annu Symp Proc.

[37] Zhang S, Bodenreider O, Golbreich C (2006). Experience in reasoning with the foundational model of anatomy in OWL DL.. Pac Symp Biocomput.

[38] Miller M, Rifaieh R (2006). Wrestling with SUMO and bio-ontologies.. Nat Biotechnol.

[39] Musen MA, Lewis S, Smith B (2006). Wrestling with SUMO and bio-ontologies.. Nat Biotechnol.

[40] Stoeckert C, Ball C, Brazma A, Brinkman R, Causton H (2006). Wrestling with SUMO and bio-ontologies.. Nat Biotechnol.

[41] Blake J (2004). Bio-ontologies-fast and furious.. Nat Biotechnol.

[42] Soldatova LN, King RD (2005). Are the current ontologies in biology good ontologies?. Nat Biotechnol.

[43] Merelli E, Armano G, Cannata N, Corradini F, d'Inverno M (2007). Agents in bioinformatics, computational and systems biology.. Brief Bioinform.

[44] Antoine Isaac JP, Daniel Rubin (2007). SKOS Use Cases and Requirements..

[45] The University of Texas Lung Cancer SPORE. P50 CA70907

[46] Baker T (2005). A Common Grammar for Diverse Vocabularies: The Abstract Model for Dublin Core.. Lecture Notes in Computer Science.

[47] Good BM, Wilkinson MD (2006). The Life Sciences Semantic Web is full of creeps!. Brief Bioinform.

[48] Neumann E (2005). A life science Semantic Web: are we there yet?. Sci STKE.

